# The RNA Helicase eIF4A Is Required for Sapovirus Translation

**DOI:** 10.1128/JVI.03174-15

**Published:** 2016-04-29

**Authors:** Myra Hosmillo, Trevor R. Sweeney, Yasmin Chaudhry, Eoin Leen, Stephen Curry, Ian Goodfellow, Kyoung-Oh Cho

**Affiliations:** a Laboratory of Veterinary Pathology, College of Veterinary Medicine, Chonnam National University, Buk-gu, Gwangju, Republic of Korea; b Division of Virology, Department of Pathology, University of Cambridge, Addenbrooke's Hospital, Cambridge, United Kingdom; c Department of Life Sciences, Imperial College London, South Kensington, London, United Kingdom

## Abstract

The eukaryotic initiation factor 4A (eIF4A) is a DEAD box helicase that unwinds RNA structure in the 5′ untranslated region (UTR) of mRNAs. Here, we investigated the role of eIF4A in porcine sapovirus VPg-dependent translation. Using inhibitors and dominant-negative mutants, we found that eIF4A is required for viral translation and infectivity, suggesting that despite the presence of a very short 5′ UTR, eIF4A is required to unwind RNA structure in the sapovirus genome to facilitate virus translation.

## TEXT

Eukaryotic initiation factor 4F (eIF4F), comprised of eIF4E, eIF4A, and eIF4G, is essential for initiation of cellular protein synthesis (1). eIF4E binds the cap structure on the 5′ end of mRNAs, while eIF4A, an RNA helicase, unwinds secondary structure in the 5′ untranslated region (UTR), facilitating ribosomal recruitment (1). eIF4G coordinates the assembly of the eIF4F complex and the recruitment of additional eIFs (1, 2).

The expression of viral proteins is frequently regulated at the level of translation initiation (2). Members of the Caliciviridae family of positive-sense RNA viruses use a novel mechanism of viral protein synthesis that relies on the interaction of initiation factors with a virus-encoded protein, VPg, covalently linked to the 5′ end of viral RNAs (3–5). The VPg proteins of feline calicivirus (FCV), murine norovirus (MNV), and porcine sapovirus (PSaV) interact with the eIF4F complex in infected cells (3, 4, 6). However, the functional roles of the components of the eIF4F complex differ among caliciviruses (3); VPg from all three viruses binds directly to eIF4E, but eIF4E is required only for PSaV and FCV viral RNA translation and infectivity, but not those of MNV (3, 4, 6).

To understand the roles of eIF4F components in sapovirus translation, we investigated the role of eIF4A in the PSaV life cycle. PSaV is a member of the Sapovirus genus of the Caliciviridae family and remains the only member of the genus capable of replication in cell culture (7, 8). PSaV is used as a model to study the mechanisms of sapovirus genome translation and replication.

**FIG 1:**
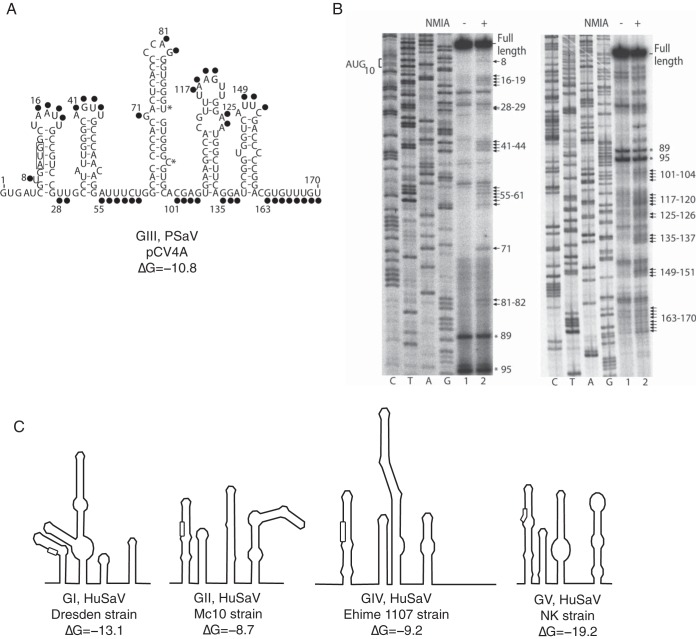
The PSaV genome contains stable stem-loops at the 5′ end. (A) Secondary structure in the 5′ end of the PSaV RNA genome predicted by Mfold (10). (B) SHAPE analysis of *in vitro*-transcribed PSaV genome (GIII, pCV4A[9]) performed as described in the text. Modified bases are indicated with an arrow. Numbers refer to nucleotide positions. The locations of the start codon and the full length are highlighted. Primers binding to nucleotides (nt) 123 to 142 (left) and 246 to 265 (right) were used. Strong bands present on both gels are marked by asterisks for reference. The positions of modified bases are marked with a black dot in panel A. (C) Predicted secondary RNA structures of Sapovirus genogroups I, II, IV, and V (Dresden, AY694184; Mc10, AY237420; Ehime 1107, DQ058829; and NK, AY646856) in schematic representation were analyzed using Mfold (10). Minimum free energies for the first stem, containing the AUG (box), in each structure are indicated as Δ*G*.

eIF4A is required for the translation of FCV and MNV (3), even though they have very short 5′ UTRs (4 to 19 bases). Using the secondary structure prediction algorithm Mfold (9), a high degree of RNA secondary structure at the 5′ end of the PSaV genome was predicted (Fig. 1A). The open reading frame 1 (ORF1) start codon is predicted to be located in the first stem, with a calculated stability of Δ*G* = −10.8 kcal/mol. The presence of five stable stem-loops (SLs) in the 5′ end was experimentally confirmed using selective 2′-hydroxyl acylation and primer extension (SHAPE) analysis. *In vitro*-transcribed PSaV RNA was modified by incubation with *N*-methyl isatoic anhydride (NMIA). Highly modified bases, characteristic of unstructured RNA, were detected by stalling in a reverse transcriptase reaction using ^32^P-labeled primers. Labeled cDNA products were separated on a denaturing 6% acrylamide gel and detected on a phosphorimager (Fig. 1B). Again, using Mfold, the start codons of other sapoviruses are shown to be similarly trapped in stems ranging in stability from Δ*G* = −8.7 to Δ*G* = −19.2 kcal/mol (Fig. 1C). The ATPase and helicase activities of eIF4A are sufficient for ribosomal scanning of 5′ UTRs with a weak to moderate secondary structure (Δ*G* = −13.1 kcal/mol or weaker) (10). The structure present in the region spanning the short 5′ UTR and the viral polyprotein coding sequences in the PSaV genome suggests that eIF4A may play a role in modifying the structure of the viral RNA to efficiently initiate virus translation (11, 12).

**FIG 2:**
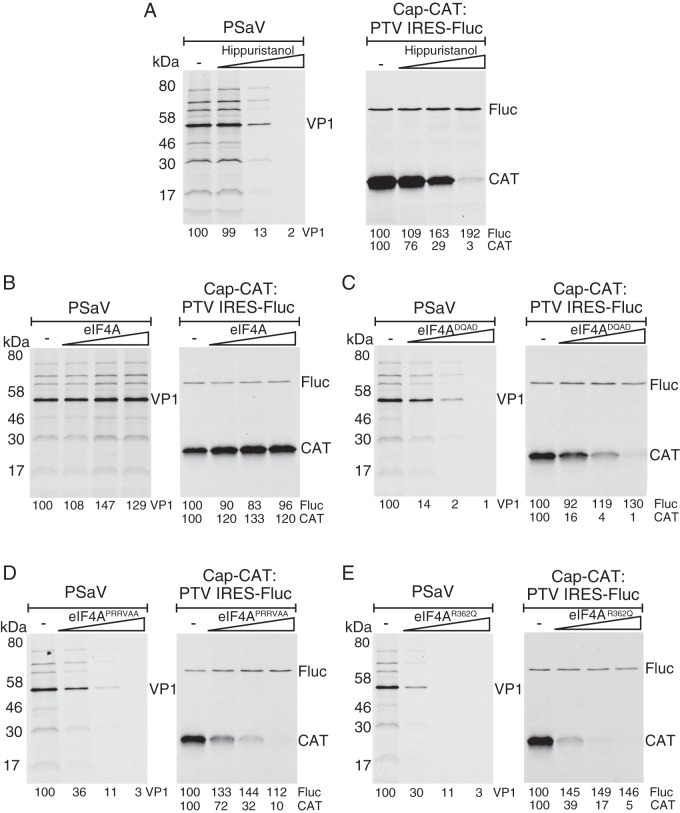
PSaV translation is inhibited by hippuristanol and eIF4A dominant-negative mutants. *In vitro* translation was performed using either VPg-linked PSaV RNA (40 ng/μl) or dicistronic RNA (20 ng/μl) containing a cap-dependent CAT and PTV IRES-dependent luciferase (Fluc). Translation reaction mixtures were preincubated with increasing concentrations of hippuristanol (A), recombinant eIF4AI wt (B), or dominant-negative mutants (C to E). RNAs were then added to initiate protein synthesis. The products for VPg-, cap-, and IRES-dependent translations were resolved by SDS-PAGE and visualized by autoradiography. The intensity of each band was quantitated with reference to the value obtained in the absence of hippuristanol, wt eIF4AI, or dominant-negative mutants as appropriate.

To characterize the role of eIF4A in the PSaV translation, the effect of hippuristanol, a specific eIF4A inhibitor (13, 14), on PSaV translation *in vitro* was examined in rabbit reticulocyte lysates (RRLs) programmed with viral VPg-linked RNA obtained from PSaV-infected cells (6). Hippuristanol is a polyoxygenated steroid that specifically inhibits the RNA binding, RNA-dependent ATPase, and helicase activities of eIF4A (13). RNA from PSaV-infected cells and *in vitro*-transcribed RNA from a dicistronic construct expressing cap-dependent chloramphenicol acetyltransferase (CAT) and porcine teschovirus (PTV)-internal ribosome entry site (IRES)-dependent luciferase were subjected to an *in vitro* translation reaction. Hippuristanol inhibited PSaV translation in a dose-dependent manner. As expected, cap-dependent CAT expression was also inhibited, but the PTV-IRES-dependent luciferase translation, which is eIF4A independent, was unaffected (Fig. 2A).

We also examined the effect of wild-type (wt) or three dominant-negative mutants of eIF4AI, E183Q (DQAD), ^109^TREL^112^→^109^RRVA^112^ (PRRVAA), and R362Q (15–17), on PSaV translation *in vitro*. While the addition of increasing concentrations of wt eIF4A slightly enhanced PSaV translation, all dominant-negative mutants inhibited PSaV translation (Fig. 2B to E). As expected, all eIF4A mutants reduced cap-dependent CAT translation, whereas eIF4F-independent PTV-IRES translation was slightly increased, possibly as a result of increased availability of other eIFs being redirected away from cap-dependent initiation (Fig. 2B to E).

**FIG 3:**
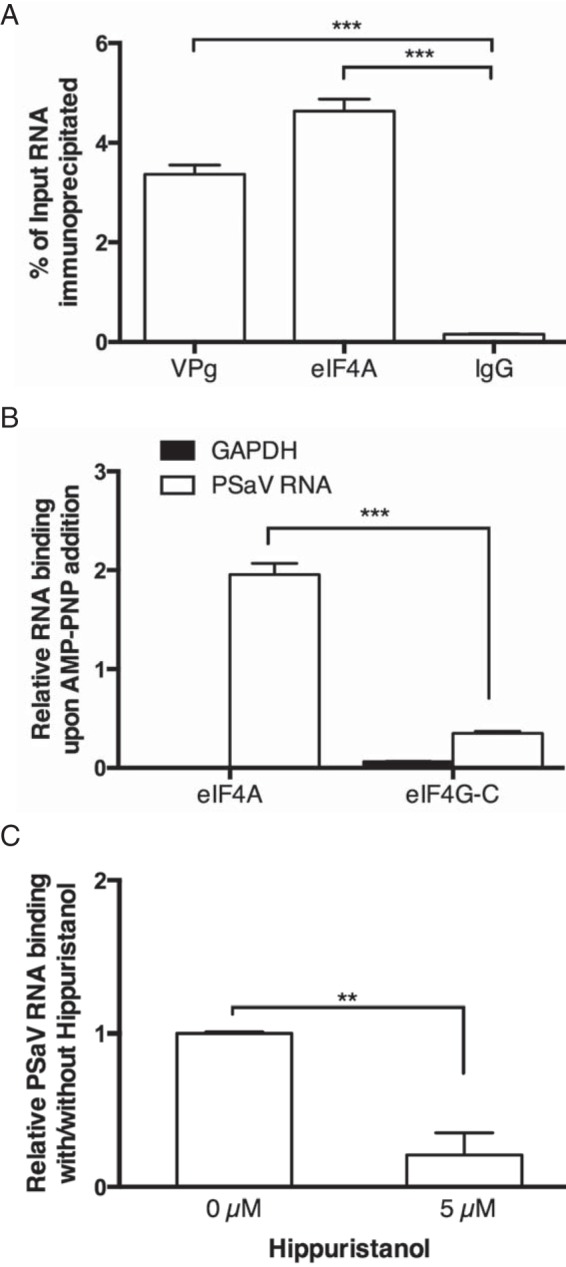
eIF4A binds PSaV mRNA during virus replication. (A) Viral RNA was coimmunoprecipitated from PSaV-infected cells with anti-VPg, anti-eIF4A, or anti-IgG antibodies. Immunoprecipitated RNA was quantified by quantitative PCR (qPCR) with primers specific for PSaV protease and is presented as a percentage relative to the total amount of input lysates. (B) Interaction between PSaV (1 μM) or GAPDH RNA (4 μM) and 10 μM eIF4AI or eIF4G-C fragment in the presence of AMP-PNP (2 mM). The His tag pulldown assay was subsequently performed with recombinant His-tagged eIF4AI or eIF4G-C. Relative RNA binding was calculated upon addition of AMP-PNP. (C) Relative RNA binding in the presence and absence of hippuristanol was assayed as in panel B. All experiments were performed three times, and the results are expressed as means ± standard errors of the means (SEM): **, *P* < 0.005; ***, *P* < 0.0001.

**FIG 4:**
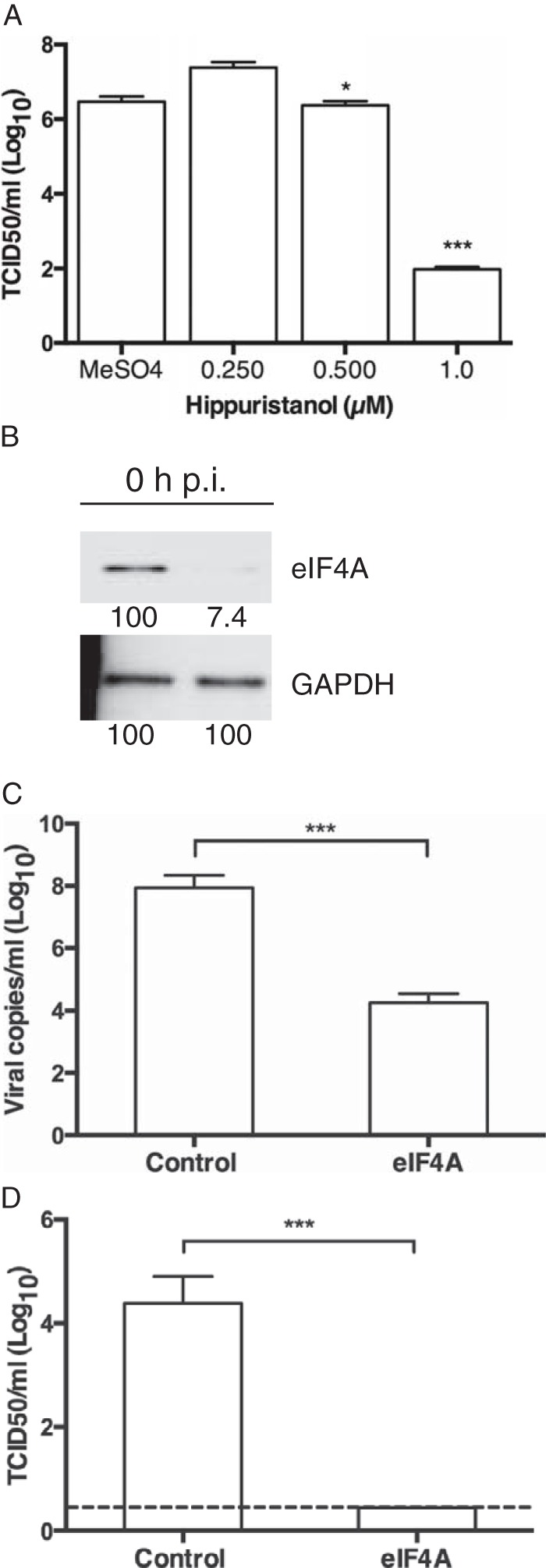
eIF4A is required for efficient PSaV replication in cell culture. (A) PSaV infectivity was determined by 50% tissue culture infective dose (TCID_50_) after treatment with MeSO_4_ or increasing concentrations of hippuristanol. (B) Transfection with either control or eIF4AI small interfering RNAs (siRNAs) was performed in LLC-PK cells. Decreased eIF4AI protein expression was verified by Western blotting prior to PSaV infection. (C) PSaV mRNA was quantitated by qPCR after control or eIF4AI siRNA transfection. (D) The levels of infectious virus were determined by titer by TCID_50_. Samples were analyzed in triplicate in three independent experiments. Error bars represent the means ± SEM from triplicate samples: *, *P* < 0.05; ***, *P* < 0.0001. The dashed line is used to indicate the limit of detection by TCID_50_.

The possibility of a direct interaction between the PSaV VPg protein and eIF4A was examined using His tag pulldown assays; however, no interaction was observed (data not shown). To investigate if eIF4A was associated with viral RNA during replication, coimmunoprecipitation of viral RNA with eIF4A was performed. Antibodies to eIF4A and VPg, as a control, were able to coimmunoprecipitate significantly more PSaV viral RNA than the control antibody (Fig. 3A). While eIF4A immunoprecipitated from infected cells could be either free or eIF4F-associated, we next examined if eIF4A could bind to the PSaV RNA directly. To examine this and confirm the specificity of interaction, recombinant His-tagged eIF4AI or the C-terminal fragment of eIF4GI (amino acids [aa] 1118 to 1600) was used to precipitate PSaV or GAPDH (glyceraldehyde-3-phosphate dehydrogenase) RNA, from RNA preparations isolated from infected cells, in the presence of the nonhydrolyzable ATP analogue adenylylimidodiphosphate (AMP-PNP), a hallmark of eIF4AI binding. eIF4AI precipitated PSaV RNA significantly, however, neither proteins precipitated GAPDH RNA (Fig. 3B), which would be expected to bind only eIF4F-associated eIF4AI. The interaction between recombinant eIF4A and PSaV RNA was also found to be sensitive to hippuristanol (Fig. 3C), consistent with the findings in Fig. 2 and Fig. 4A, confirming a direct specific interaction between eIF4A and the PSaV genome.

To examine the functional role for eIF4A in the PSaV life cycle, the effect of hippuristanol on PSaV replication in cell culture was examined. The PSaV titer was reduced by hippuristanol in a dose-dependent manner (Fig. 4A) at concentrations where cell viability was unaffected (data not shown). This suggests that PSaV VPg-dependent translation may be more susceptible to eIF4A inhibition than canonical cellular translation, although potential contributions from pleotropic effects of the drug cannot be excluded. Depletion of eIF4AI by RNA interference (Fig. 4B) resulted in significantly reduced PSaV genome levels and yields of infectious virus (Fig. 4C and D). Together with our *in vitro* analysis, our results demonstrate a functional role for eIF4A in the PSaV life cycle, providing additional insight into the novel mechanism of protein-primed translation initiation and the life cycle of poorly characterized caliciviruses.
